# Operation of Tracheotomy in an Epileptic

**Published:** 1852-09

**Authors:** 


*Operation of the Tracheotomy in an Epileptic.* By Dr. Neill.—The views of Marshall Hall, which have lately appeared in the English journals upon the subject of epilepsy, have probably fallen under the notice of most of the Fellows of the College.

Every investigation of a malady so distressing, and of which so little has been known of its pathology, must be hailed with pleasure by every practitioner of medicine. Especially will this be so, when so high an authority upon affections of the nervous system advances views not only as to the nature of the disease, but also practical deductions of the highest importance.

Dr. Marshall Hall says: "This question of the application of tracheotomy in the preventive treatment of epileptic convulsion, is one involving high principles in physiology.

"As I have stated, I believe few will hesitate to perform the operation of tracheotomy, as the present remedy, when there is, from apoplectic laryngismus, imminent danger to life. But the question remains—are we justified in performing this operation in cases of epileptic and other convulsions, as a preventive of future evil? Are the somewhat remoter dangers to mind, and limb, and life, and the hope that whilst the faculties are spared the patient may be rescued from the susceptibility to the attacks, the *dignus vindice nodus*, a sufficient motive for adopting this measure in its more continuous mode of a tube worn in the trachea. After having witnessed the dire circumstances and effects of the frightful maladies more than any man, of epilepsy especially, I unhesitatingly say, yes! I regard the melancholy condition of the patient as justifying the heroic remedy. The case may be violent and frightful *in any degree.* In what precise case tracheotomy is justifiable I do not pretend to determine. It is a matter of pure *moral* calculation and choice in regard to the terrors of the malady on one hand, and of the remedy on the other. Epilepsy may occur in the slightest form of mere transient oblivion, and it may occur in the gravest form of sudden and violent convulsion, dashing the patient to the ground, into the fire, or into the water, and followed by coma or apoplexy, delirium or mania, paralysis, amentia.

"The former of these attacks may be designated the *epilepsia mitior.* Itcomprises all that is short of laryngismus, affections of the senses, as muscæ, tinnitus, the odor of musk, aura, vertigo, oblivion, confusion, loss of consciousness, nutatio, falling, various spasmodic affections of the face, the eyes, the extremities.

Then comes laryngismus, laryngeal dyspnœa, perhaps perfect closure of the larynx, with violent efforts of expiration. This with all the other links of the dreadful chain constitute the *epilepsia gravior;* all that is on *this* side of the laryngismus must be unaffected by the operation of tracheotomy; all that is on *that* side of this laryngismus will, I trust and believe, be prevented by its efficient institution. By tracheotomy, the *epilepsia gravior* or the 'grand mal,' is converted into the *epilepsia mitior*, or the 'petit mal.' If this, my hope, be realized, I shall deem the event a great victory achieved by physiology or theory over mere observation, and especially by that of the diastaltic nervous system, of which it is an application.

"I may now observe, in conclusion, that I have on several occasions stated that, if tracheotomy were performed, and a tube worn in the trachea, the epileptic, the puerperal, or the infantile convulsion would be prevented, with its dire effects."

In accordance with these views I operated upon a patient of Dr. Shelmerdine, in Spring Garden, under whose care he had been for about one year, and who had tried all the ordinary modes of treatment. The following are the particulars of his case:—

John Blume, aged twenty-nine, of five feet eleven inches in height, and weight about one hundred and sixty pounds. His appearance was healthy, and he had no deformity of the throat.

His first fit occurred nine years ago, and was not referable to any particular cause by his family. He was not subject to them in childhood, although his brother had died of epilepsy.

The frequency of the paroxysms gradually increased, and for the last year he has been unable to attend to any business.

His mind has been so affected by the disease that he has frequently mistaken his way home, and often gone into the neighbors' houses for his own.

His mother and wife informed me that during the last six months he would have an attack at least every other day, but occasionally would have as many as fifteen or twenty during the day.

Life had become a burden to him, and he feared to leave his home.

His physician tells me that on the first occasion of his being called to him, he was laboring under most severe congestion of the face and neck, producing great lividity and complete insensibility; and that, in all of the subsequent attacks, difficulty of breathing seemed to be prominent.

The patient himself remarked to me that, immediately preceding his attacks, he frequently experienced a sense of constriction about his windpipe; and his friends and family confirmed the idea, that the severity of the attack was proportionate to the difficulty of breathing.

When the operation was proposed, and its nature explained to him, he was anxious for its performance, and had great expectations of its relieving him.

The operation was performed on the 11th of March last, in the presence of Drs. Shelmerdine, Marshall Paul, and Hollingsworth.

His neck was long and well adapted for the operation. The incisions were made in the usual way, and the only points worthy of remark were—that the sterno-hyoid muscles, from frequent spasmodic contractions, were thicker than usual; and that the isthmus of the thyroid gland was so large and broad as to cover the first three rings of the trachea.

The hemorrhage was not so troublesome as might have been expected ; care was taken to tie the inferior thyroid vein, and no irregular artery was met with after the trachea was exposed. A piece of about three lines in breadth, was removed from the middle of the fourth ring of the trachea, and the fifth ring also was divided in order to accommodate more accurately the tube which had been provided, which was of the ordinary form of the instrument shops.

The introduction of the tube produced but little irritation and coughing, his voice was not in the least affected ; but the trachea was smaller than usual, and the wound becoming so very deep after the division, that I had constructed tubes of various angles and length corresponding with the depth of the wound. (Specimens of the tubes were exhibited to the College.)

He slept but little the first few nights after the operation, and seemed unwilling at first to trust himself in a recumbent position, but as the wound healed around the tube he became comfortable, and had nothing like a return of his complaint until the *thirteenth* day after the operation, which tendency to an attack he attributed to his removal of the tube; he had taken a slight cold, which made the tube disagreeable on that day, and he thought he would risk the night without it. The spasm was slight, and he did not lose his consciousness. About *two weeks* after this he was threatened with an attack of which he was conscious, and mentioned the fact to his mother, who immediately removed a temporary plug which he introduced in the orifice of his tube to prevent a whistling noise accompanying respiratory movements. Upon the removal of the plug, the symptoms disappeared, his breathing was comfortable, and he felt much encouraged. He began to appreciate the object of the operation, and fully believed that the means to mitigate the severity of his attacks was the removal of the plug, and that the disease was under his own control.

He made arrangements to renew his business, walked about the streets in the confidence and consciousness of a strength of mind and purpose which he had not experienced for a long period.

Unfortunately for him, however, he was again seized on the evening of the 2d of May, with symptoms of another attack. His physician was sent for, who removed the tube and cleansed it; after it was replaced the patient felt easier, but was not completely relieved. In the middle of the night he had a most violent attack, and died almost instantaneously. His physician was not with him when he died, and the family would not permit a *post-mortem* examination of any part of his body but his throat. Dr. Shelmerdine merely examined the cicatrix around the wound and trachea. The parts had consolidated around the tube, and the trachea was perfectly healthy.

I report this case to the College in order that they may form their own judgment upon the theory and the treatment of Marshall Hall. Few cases have as yet been reported where this operation has been performed, and I believe that this is the first case in this country in which the trachea has been opened, and a tube worn, in order to mitigate, if not prevent, attacks of epilepsy.

And, although this patient died, I still think favorably of the operation, and under the same circumstances would perform it again. His death was in no way attributable to the operation, and had not the operation been performed it might have occurred at a still earlier period. I regard the mitigation of the attacks with which he was once threatened, and moderation of the symptoms, as more satisfactory than if there had been no approach of an attack, for then the entire absence of the complaint might have been attributed to the shock made upon the system by the operation; and this operation would have demonstrated nothing more than tying the carotid artery, after which, and other violent shocks, patients have been free from attacks for a long period.—*Transactions of the College of Physicians of Philadelphia.*

